# Testing the effect of summer camp on excess summer weight gain in youth from low-income households: a randomized controlled trial

**DOI:** 10.1186/s12889-020-09806-y

**Published:** 2020-11-17

**Authors:** E. Whitney Evans, Rena R. Wing, Denise F. Pierre, Whitney C. Howie, Morgan Brinker, Elissa Jelalian

**Affiliations:** 1 Weight Control and Diabetes Research Center, The Miriam Hospital, 196 Richmond St, Providence, RI 02903 USA; 2 Department of Psychiatry and Human Behavior, Warren Alpert Medical School, Providence, RI USA; 3 Institute for Collaboration on Health, Intervention, and Policy, University of Connecticut, Hartford, CT USA; 4 Department of Psychological & Brain Science, Texas A&M University, College Station, TX USA; 5 School of Public Health, Brown University, Providence, RI USA

**Keywords:** Childhood obesity, Physical activity, Diet, Sedentary behavior

## Abstract

**Background:**

Children from racial and ethnic minority groups, low-income households, and those with overweight or obesity gain more weight during the summer than the school year. Summer day camps, which offer routine opportunities for physical activity and regular meal and snack times, have potential to mitigate excess weight gain. This randomized controlled trial was done to determine the feasibility and preliminary effectiveness of summer camp in preventing excess summer weight gain among youth from low-income households.

**Methods:**

Children, ages 6 to 12 years, were randomized to attend 8-weeks of summer day camp (CAMP) or to experience an unstructured summer as usual (SAU) in 2017–2018. Primary feasibility outcomes included retention, engagement and completion of midsummer measures. Secondary outcomes included changes in BMIz, engagement in moderate to vigorous physical activity (MVPA) and sedentary behavior, and diet quality and energy intake from the school year to summer. Multivariable linear mixed models were used to assess group differences.

**Results:**

Ninety-four participants were randomized to CAMP (*n* = 46) or SAU (*n* = 48), of whom 93.0 and 91.6% completed end of school and end of summer assessments, respectively. While CAMP participants attended only 50% of camp days offered, on average, they lost − 0.03 BMIz units while those in SAU gained 0.07 BMIz units over the summer (b = 0.10; *p* = .02). Group differences in change in energy intake from the school year to summer were borderline significant, as energy intake remained relatively unchanged in CAMP participants but increased among participants in SAU (*p* = 0.07).

**Conclusions:**

Randomizing children to attend summer day camp or experience an unstructured summer as usual was effective in this low-income sample. Our findings support the potential for summer camps in mitigating excess summer weight gain. A larger randomized trial is needed explore efficacy, cost-effectiveness and longer-term effects of attending summer camp on weight and weight-related behaviors.

**Trial registration:**

ClinicalTrials.gov Registration: NCT04085965 (09/2019, retrospective registration).

## Background

Childhood obesity disproportionately affects youth from low-income households. The prevalence of obesity is nearly twice as high in children from low-income versus higher income households (18.9% vs. 10.9%) [1]. These disparities often persist through the life course as youth with overweight or obesity are more likely to have adult obesity and are at increased risk for developing diabetes and cardiovascular disease risk factors [2–4]. Since the majority of American children spend 6 to 7 h per day at school, schools have been a priority setting for addressing disparities in obesity prevalence [5]. However, despite significant progress improving access to healthy nutrition and physical activity within schools, disparities continue to widen [1]. New evidence suggests that this might be because school-based interventions neglect the critical period of summer.

Longitudinal data show that children, particularly those from low-income communities, racial and ethnic minority groups, and those with overweight and obesity, gain more weight during the 3 months of summer than during the nine-month school year [6–10]. While the causes of excess summer weight gain are not fully understood, the Structured Days Hypothesis suggests that in the absence of the routine and structure provided by the school day, children have greater engagement in obesogenic behaviors [11, 12]. During the summer, children lose access to routine physical activity opportunities provided by physical education and recess and calorie-controlled, nutritionally-balanced school meals. As a result, those without structured summer plans engage in fewer minutes of moderate to vigorous physical activity (MVPA), spend more time sedentary, lose physical fitness gains achieved during the school year, and consume a lower quality diet [13–15]. Thus, in their 2019 report, *Shaping Summertime Experiences: Opportunities to Promote Healthy Development and Well-Being for Children and Youth*, the National Academies of Sciences recognizes summer as a vulnerable period and calls for research to inform best practices to safeguard children’s health and wellbeing over the summer [16].

Residential summer camps have been used successfully to promote weight loss in youth with overweight or obesity; however, less has been done to examine the effectiveness of summer day camps in obesity prevention [17–25]. With over 5000 summer day camps available across the US, they represent a mode of intervention with high potential for dissemination [26, 27]. Moreover, given that typical summer day camps provide structure and include physical activity and meal provision, they are well positioned to address excess summer weight gain. Data from community-based summer camps show that on days they attend camp, 80% of boys and 73% of girls meet the daily recommendation of 60 min of MVPA [28]. Similarly, children are significantly more active on weeks they attend summer day camps as compared to when they are home [14]. While these and other studies [29] suggest that camp participation may impact physical activity engagement, few studies have examined the effect of summer day camps on excess summer weight gain among youth from low-income households.

The primary objective of this pilot randomized controlled trial was to examine the effectiveness of randomizing children, ages 6–12 years from low-income communities, to attend summer day camp (CAMP) or to experience summer as usual (SAU). Primary Analyses included testing multiple aspects of study feasibility including: i) retaining participants in a randomized controlled trial over the summer, ii) adherence to randomization assignment (i.e. CAMP children attending camp and SAU participants having an unstructured summer, or one that included 1 week or less of structured summer programming), and iii) data collection on weight-related behaviors over the summer. Secondary analyses examined measures of preliminary effectiveness including comparing group differences in changes in BMIz, percent time spent in MVPA or sedentary behavior, total energy intake and dietary quality from the end of the school year to the summer. We hypothesized that relative to those randomized to SAU, children randomized to CAMP would experience less excess summer weight gain and spend more time active and less time sedentary and consume a lower energy, higher quality diet.

## Methods

### Study design

This randomized controlled trial was carried out in two low-income communities in the Northeast during summers 2017 and 2018. Children were randomized in a 1:1 fashion to CAMP or SAU using a randomization schedule generated by the study co-PI using Proc Plan in SAS 9.4 (2014; SAS Institute, Inc., Cary, NC). Sibling pairs were randomized as a unit. Summer day camp was provided by the Boys and Girls Club in each community, and all camp fees were covered by the research study. This study was funded by the Hassenfeld Child Health Innovation Institute at Brown University, adheres to CONSORT guidelines for a randomized controlled trial and was approved by the Institutional Review Board at Rhode Island Hospital.

### Participants

Participants, ages 6–12 years, were recruited through the local housing authority, the public school district and via community events. Flyers inviting families to participate were mailed to housing authority residents and sent home via backpacks. Interested families were invited to attend an enrollment visit. To enroll, children had to 1) qualify for free or reduced-price meals at school, 2) speak English (for purposes of camp participation), and 3) agree, along with their parent(s), to randomization. Participants were excluded if they had a medical condition that interfered with participation in physical activity or if they were otherwise enrolled in summer programming (camp, summer school, etc.) for more than 1 week. Parental informed consent was obtained for all children enrolled in the study. Child assent was obtained from those > 8 years. Parents / guardians received an honorarium at baseline and end of summer for completing assessments.

### Camp

Children randomized to CAMP were enrolled in 7- (2017) or 8-weeks (2018) of day camp offered by local Boys and Girls Clubs (BGC) in each community. In 2017, only 7 weeks of camp were offered as the school year ran longer due to snow day make-ups. BGC camps in both communities were offered daily from 8:30 AM to 4 PM. The BGC provided daily transportation from each housing community to camp. Children were grouped by age (6–8 years, and 9–12 years) and assigned to a counselor. Each day, counselors led campers through activities including sports, games, obstacle courses, swimming and boating, and arts and crafts in 45–60 min blocks. Free breakfast and lunch meals were provided to campers daily via the USDA’s Summer Food Service Program (SFSP) [30]. Per federal guidelines, SFSP meals must include 8 oz. of milk, ¾ cup of fruit / vegetable, 1 serving of grains / breads, and 1 serving of lean protein or equivalent [31].

### Summer as usual

Participants randomized to SAU were asked to experience summer vacation as otherwise planned by their parent/guardian. As part of the consent process, they confirmed that they were not enrolled nor planned to enroll in a summer day camp or other daily structured summer programming (i.e. summer school or day care) for more than 1 week over the summer.

### Assessment schedule & outcome measures

All participants completed study assessments with trained research staff, blind to randomization, at the end of the school year (baseline), during weeks four and five of the 8-week summer (midsummer), and during the last week of summer (end of summer). At baseline, each child was weighed and measured, and a parent / guardian completed a sociodemographic questionnaire, which included questions on participant age, sex, race/ethnicity, and maternal education. In summer 2018, parent / guardian also completed a caregiver questionnaire.. At baseline and midsummer, participants completed three 24-h diet recalls and wore an ActiGraph for 24-h per day for 1 week. Finally, at end of summer, the child was weighed and measured a second time. After completing the baseline assessment, participants received a sealed envelope from the study coordinator, which disclosed their randomization.

#### Change in BMIz

Height and weight were measured in triplicate at baseline and end of summer. Child weight was measured, without shoes, to the nearest 0.1 kg using a calibrated digital scale (Tanita BWB 800; Tanita Corporation of America, Inc., Arlington Heights, IL). Height was measured to the nearest millimeter using a portable stadiometer (Model 214, Seca North America, Chino, CA). BMI-for-age percentile and BMI-for-age z-scores (BMIz) were calculated using the Centers for Disease Control and Prevention (CDC) standards [32]. Weight categories were defined according to CDC cut points for age and sex [33]. Change in BMIz from the school year (baseline) to end of the summer was used as a proxy for excess summer weight gain.

#### Dietary intake

Diet was assessed at baseline and midsummer via three, non-consecutive 24-h diet recalls (2 weekdays, 1 weekend day). Registered dietitians or graduate level nutrition students collected the recalls over the phone using Nutrition Data Systems for Research (NDSR; Nutrition Coordinating Center, University of Minnesota, Minneapolis, MN). NDSR uses a variation of the USDA’s validated Automated Multiple Pass Method to collect detailed information on each food and beverage consumed at each eating occasion over the previous day [34]. Participants 9 years and older completed the recalls with parent / caregiver input as needed, while proxy-assisted interviews were conducted for participants ages 6–8 years. More specifically, in a proxy-assisted interview, the parent / caregiver and child complete the recall together so that the child can assist in reporting intake information [35]. NDSR output was used to calculate average reported energy intake and Healthy Eating Index, 2015 (HEI-2015) total scores for each participant at baseline and midsummer. The HEI-2015 is a density-based measure of diet quality that assesses adherence to the Dietary Guidelines 2015–2020 [36, 37].

#### Physical activity & sedentary behavior

Percent time spent in moderate to vigorous physical activity (MVPA) or in sedentary behaviors were measured for 24-h per day for 1 week at baseline and midsummer using a wrist-worn accelerometer (wGT3X-BT, ActiGraph LLC, Pensacola FL). Participants wore the ActiGraph on their non-dominant wrist for 24-h to improve adherence [38]. Using ActiLife software, participant actigraphy data were considered valid and included in the analyses if daily wear time was > 8 h on a minimum of four weekdays [39]. Given our interest in how attending camp or having unstructured days during the week affects changes in MVPA, we only assessed activity behaviors on weekdays. The Chandler et al vector magnitude regression cut-points for wrist-worn accelerometry, which apply 60 s epochs, were applied to define percent time in MVPA and sedentary behaviors [40].

#### Participation

Each week throughout the summer, primary caregivers completed a participation survey via text message or phone, which captured data on their child’s daily participation in the BGC camp or other structured summer programming (summer school, other summer camps, or the SFSP). Daily camp attendance data were also collected from the BGC camps at the end of each summer.

#### Caregiver status

In 2018 only, parent / guardian completed a questionnaire at baseline indicating their child’s primary caregiver and his/her employment status (unemployed, employed part-time, employed full-time).

### Data analyses

All statistical analyses were conducted using SAS 9.4, at the two-tailed 0.05 level of significance. General descriptive statistics were generated for demographics and anthropometrics at baseline. Group differences were assessed using student’s t-tests or Chi-square tests as appropriate. To address our primary aim to assess the effectiveness of randomizing children to attend summer day camp or SAU, we evaluated process measures of intervention dose and assessment completion using descriptive statistics. This trial was not powered a priori to test for intervention effects on BMIz and weight-related behaviors; however, to inform preliminary effectiveness of attending summer day camp, we examined group differences in BMIz (excess summer weight gain), minutes of MVPA, percent time spent sedentary and diet (total energy intake and diet quality). Separate linear mixed models with maximum likelihood estimation, to account for the correlation among siblings randomized as pairs, were used to estimate group differences in dependent variables including change in BMIz, minutes of MVPA, percent time spent sedentary, and diet measures. The primary independent variable was random group assignment, and each model accounted for clustering by family and controlled for baseline values, year of participation, age and race/ethnicity. Linear mixed models used to analyze longitudinal change in these outcomes required follow-up measures, such that only those participants with complete data are included these secondary analyses. Separately, we also tested for effect modification by baseline level of overweight / obesity and year (2017 vs. 2018) to determine if either moderated the effect of group assignment on excess summer weight gain. We did so by testing the significance of an interaction term.

## Results

**Fig. 1:**
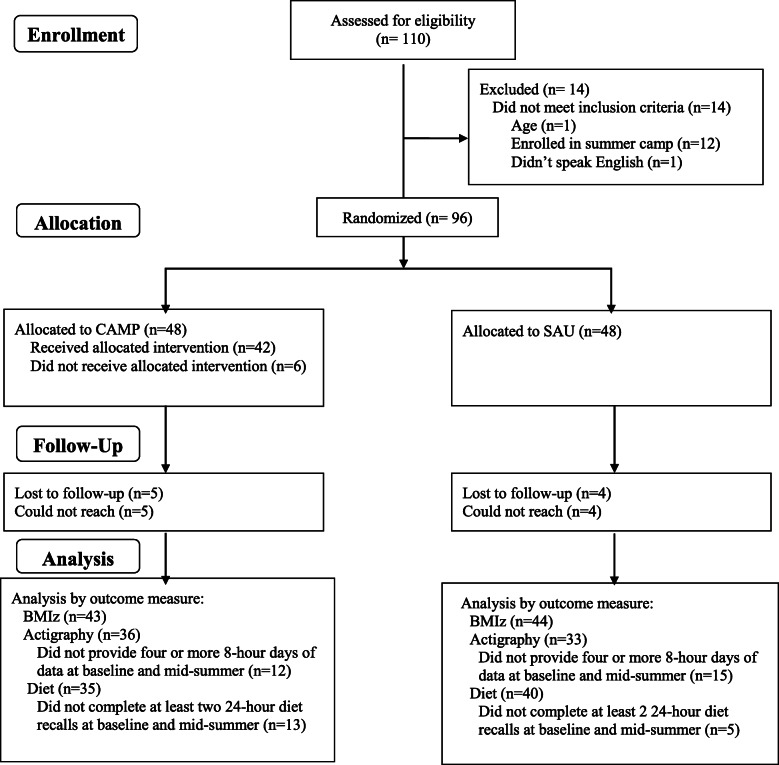
Consort diagram

As shown in Fig. 1, a total of 96 participants enrolled in this study and were randomized to CAMP (*n* = 48) or to SAU (*n* = 48). In year one, a total of 40 children were enrolled and randomized, and in year 2, 56 children were enrolled and randomized. Post-hoc power analyses for group differences in BMIz suggest that with a sample size of 94 and group difference in BMIz of 0.1 + 0.04, we had greater than 90% power. Two participants randomized to CAMP never completed the BGC camp enrollment process and were then lost to follow-up. Table 1 shows the baseline characteristics of each group. On average, participants in both groups were 9 years old and were predominately from racial / ethnic minority groups. Participants in CAMP were 58.3% female versus 43.8% in SAU, but this difference was not statistically significant (*p* = 0.15). In both groups, nearly half of the participants had mothers with a high school degree or less (51.8% in SAU and 48.3% in CAMP, *p* = 0.67), and in both groups, more than half met criteria for having overweight / obesity at baseline (53.2% in SAU and 60.9% in CAMP; *p* = 0.45).

**Table 1:** Characteristics of children, ages 6–12, randomized to experience an unstructured Summer as Usual (SAU) or attend a summer day camp (Camp)

	SAU (*n* = 48)	Camp (*n* = 46)	***p***-value
Age *(mean years (SD))*	9.08 + 1.81	8.87 + 1.93	0.59
Summer 2017 participant (%)^a^	41.7	40.8	0.93
Female (%)	43.8	58.3	0.15
Race/Ethnicity (%)			
Non-Hispanic White	11.1	6.4	0.87
Non-Hispanic Black	15.6	14.9	
Non-Hispanic Other	13.3	12.7	
Hispanic, All Races	60.0	66.0	
Maternal Education of High School Degree or Less (%)	51.8	48.3	0.67
Qualification for Free Lunch (%)	93.8	93.8	1.0
Overweight / Obesity^b^ (%)	53.2	60.9	0.45

^a^Participants were recruited in Summers 2017 and 2018. This row specifies the breakdown of enrollment and randomization by year

^b^Overweight / Obesity determined by BMI for age and sex >85th percentile

### Participation

CAMP attendance averaged 15.8 + 7.7 of 34 days offered in 2017 (46%), and 21.0 + 10.2 of 39 days offered in 2018 (54%). Across the 2 years, participation ranged from 2 to 37 days with standard deviation of 10.2 days. In 2018, when caregiver employment was assessed, camp attendance was related to caregiver employment, as those with a caregiver employed full-time attended 27 + 5.9 (69.2%) days of camp, while those whose caregiver was employed 20 h per week or less attended 14.6 + 14.7 (37.4%) days of camp (*p* = 0.02). Among SAU participants, parents / guardians reported that their children only attended 1.2 days of camp or structured summer programming (i.e. summer school) over the summer.

### Changes in BMIz

**Fig. 2:**
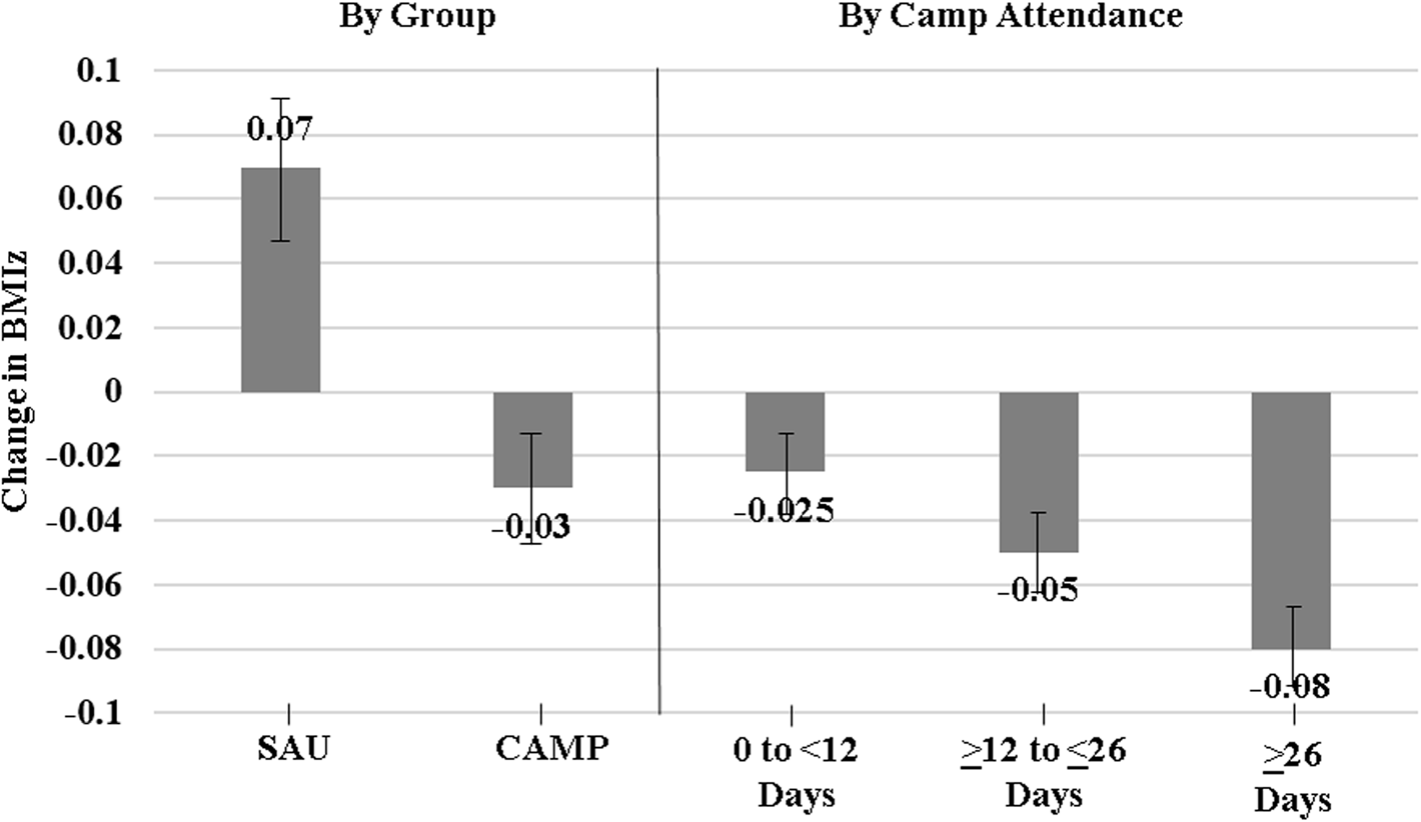
Change in BMIz over the summer by group (SAU vs. CAMP) and by camp attendance within CAMP

Forty-four participants randomized to SAU (91.6%) and 43 of those randomized to CAMP (93%) completed both the baseline (end of school year) and the end of summer assessments. As shown in Table 2, On average, participants randomized to camp experienced a decrease of − 0.03 BMIz units, while BMIz in those randomized to SAU increased by + 0.07 BMIz units (group difference in change in BMIz between SAU and CAMP, b = 0.10; *p* = .02). Moreover, there was no significant effect modification by year (*p* = 0.97) nor baseline weight status (*p* = 0.14). Given an average attendance of 50% across the 2 years, we examined the relationship between change in BMIz and camp attendance among CAMP participants. BMIz decreased, on average, by 0.004 units for each additional day of camp participation over the summer (b = − 0.004, *p* = 0.06). Figure 2 depicts change in BMIz over the summer by group (SAU vs. CAMP) and by camp attendance within CAMP.

**Table 2:** Changes in relative weight and weight-related behaviors from the school year (baseline) to midsummer among children randomized to attend a daily summer camp or to experience summer as usual (SAU)

	SAU	CAMP	Group Difference (effect estimate; ***p***-value)
***Change in BMI z-score (BMIz)*** *(n = 44 in SAU; n = 43 in CAMP)*			
School^a^	1.25 + 0.17	1.43 + 0.18	
Mid-Summer^a^	1.34 + 0.19	1.41 + 0.19	
Change^b^	+ 0.07 + 0.03	−0.03 + 0.03	b = 0.10; *p* = .07
***Change in percent time spent in Moderate to Vigorous Activity*** *(n = 33 in SAU; n = 36 in CAMP)*			
School^a^	9.7 + 0.6%	9.4 + 0.6%	
Mid-Summer^a^	8.1 + 0.8%	7.5 + 0.8%	
Change^b^	−1.9 + 0.7%	2.3 + 0.7%	b = 0.40; *p* = .65
***Change in percent time spent Sedentary*** *(n = 33 in SAU; n = 36 in CAMP)*			
School^a^	61.8 + 1.1%	62.3 + 1.2%	
Mid-Summer^a^	65.9 + 1.7%	66.8 + 1.8%	
Change^b^	+ 4.7 + 1.6%	+ 5.3 + 1.7%	b = − 0.64; *p* = .75
***Change in Energy Intake*** *(n = 40 in SAU; n = 35 in CAMP)*			
School^a^	1495.9 + 91.6	1519.1 + 98.0	
Mid-Summer^a^	1682.8 + 165.4	1464.3 + 171.0	
Change^b^	+ 247.8 + 130.4	−52.5 + 135.4	b = 300.3; *p* = .07
***Change in Dietary Quality (HEI-2015 total score)*** *(n = 40 in SAU; n = 35 in CAMP)*			
School^a^	47.9 + 2.0	46.9 + 2.2	
Mid-Summer^a^	48.6 + 1.9	48.5 + 1.8	
Change^b^	+ 0.49 + 1.8	+ 0.12 + 1.9	b = 0.4; *p* = .87

^a^Adjusted means from linear mixed model adjusted for clustering by family and controlling for year, age and race/ethnicity

^b^Adjusted means from linear mixed model adjusted for clustering by family and controlling for year, age, race/ethnicity and baseline values

### Behavior changes

We examined group differences in change in MVPA, percent time spent sedentary, total energy intake and diet quality between the end of the school year and midsummer. Valid accelorometry data were provided by 33 (69%) participants in SAU and 36 (75%) CAMP participants at baseline and midsummer. Forty (83%) and 35 (76%) provided usable dietary data in SAU and CAMP, respectively, at both time points. As shown in Table 2, there were no statistically significant group differences in change in percent time spent in MVPA, sedentary behavior or diet quality from the school year to summer (p’s > .05). There was a trend toward significant group differences in change in reported total energy intake (*p* = .07). Specifically, reported total energy intake remained similar at baseline and midsummer in CAMP participants and increased in SAU participants (difference in group changes b = 334 kcals/day; *p* = .07).

## Discussion

Findings from this pilot randomized controlled trial suggest that when children from low-income households are randomized to attend summer day camp or to experience an unstructured summer as usual, they adhere to their randomization assignment. More specifically, children randomized to SAU did not enroll in other summer camps or structured programming. In addition, preliminary evidence indicates that children randomized to CAMP experience smaller changes in BMIz relative to those randomized to SAU (− 0.03 BMIz units vs + 0.07 BMIz units, *p* = .02). Given that there are over 5000 summer day camps available to children across the US [27], they have significant potential to help prevent excess summer weight gain.

The majority of studies examining summer programming (camp or otherwise) have not been randomized controlled trials with an inactive control group. In this study we were able to use the stronger randomized trial design to analyze the effects of CAMP vs SAU on summer weight changes, to recruit children from low-income families, and to complete baseline, midsummer, and end of summer assessments on significant proportions of these children. Specifically, more than 92% of participants were retained in the study across the two summers, and the majority of participants also completed midsummer assessments. Despite the BGC providing transportation to / from camp each day, however, CAMP attendance averaged 50% across the two summers. While lower than expected, attendance was similar to that observed in Camp NERF, an 8-week, multicomponent camp offered with the SFSP, in which participants attended 56.8% of offered sessions [41]. Moreover, CAMP attendance was related to change in BMIz over the summer and trended higher among children whose primary caregiver was employed full-time. This finding speaks to the potential for the use of childcare vouchers in helping summer camps to help address weight related health disparities. Government-funded childcare vouchers are provided to working parents from low-income households, so while the ability to use them to cover summer day camp tuition costs varies by state across the US, [42] they increase the potential of summer camps to help address weight-related health disparities in low-income working families. However, qualitative research is needed to better understand barriers to and motivators for attending camp when tuition is covered, so that camps can respond and maximize attendance.

To our knowledge, this is the first randomized control trial to suggest that a nationally available summer day camp model mitigates excess summer weight gain when compared to an inactive control group. Other studies examining summer programming have likewise suggested summer day camp has beneficial effects. In *Healthy Lifestyle Fitness Camp*, a quasi-experimental study examining the effectiveness of a 6-week summer program that included 3 h of daily physical activity programming, nutrition education and lunch through the SFSP, George and colleagues found that intervention participants lost 1.3 kg over the summer, while those in the control group gained 0.31 kg (*p* < .001) [43]. Similarly, in our own quasi-experimental study, *Promoting Health and Activity in the Summer Trial*, we tested the effectiveness of an 8-week, half-day activity-based program that included lunch provided through the SFSP and found that intervention participants lost 0.04 BMIz units, while those in the control group gained 0.03 units (*p* = .06) [24]. Again, as in this study, attendance at the summer camp program was related to the magnitude of weight gain over the summer.

Our findings preliminarily suggest that summer day camp may prevent increases in total energy intake during the summer relative to children who experience an unstructured summer. Given that the BGC provide lunch via the SFSP, which is governed by the CACFP nutrition guidelines [31], we anticipated that children who attended camp would maintain better dietary quality from the school year to the summer relative to those in SAU. Our preliminary data do not support a substantial change in diet quality between the school year and summer nor group differences. Instead, they suggest that children in both groups consumed a low-quality diet at both time points (49 out of a possible 100), [44] which is consistent with national average of 52.0 points in children ages 6–11 years [45].

The majority of studies examining the effect of summertime interventions on excess weight gain have looked at changes in physical activity and sedentary behavior. Similar to observational research [46], our findings suggest that engagement in MVPA decreased and percent time spent sedentary increased from the school year to the summer; however, unexpectedly, we found no group differences in either measure. It is possible that our null findings are attributable to low camp attendance during the 7-day midsummer assessment period, as Weaver and colleagues found that children attending a summer day camp were significantly more likely to meet the daily recommendation for 60 min of MVPA as compared to children not attending camp [47] and similar findings were reported for the *Girls in the Game* summertime intervention [25, 48]. Unfortunately, we are unable to examine MVPA engagement on days we know children attended camp, as attendance data were provided in aggregate by the BGC camps.

This study has strengths and limitations. Strengths include the fact that the trial was conducted in racially / ethnically diverse samples in which the prevalence of overweight / obesity in youth was very high. Additionally, a randomized trial design was used and comprehensive measures of dietary intake and physical activity were completed at baseline and mid-summer (i.e. when the children were participating in the camp experience). Finally, we partnered with BGC, a community-based organization that offers day camps with similar structure and program across the United States [49]. Limitations of this study include that it was a pilot study conducted across two communities, for which post-hoc power analyses were completed. Although our analyses suggest that randomization to camp may mitigate excess summer weight gain relative to an unstructured summer, a larger randomized controlled trial is needed. Second, average camp attendance was 50% over the two summers, which was lower than anticipated. This may be attributable to caregiver employment and the availability of alternate care; however, better understanding of variables related to attendance at summer structured activities is needed. Finally, we had participants wear the ActiGraph monitors on their wrists to increase adherence; however, wrist placement may inflate MPVA estimates and there is no agreement on wrist-worm cut points for analysis [50]. To account for this, we compared wrist-worn data collected at the end of the school year and mid-summer, as we would expect that the inflation would be comparable in both groups.

## Conclusions

Findings from this pilot randomized controlled trial support the feasibility of randomizing children from low-income households to experience summer day CAMP or an unstructured SAU and of collecting data on weight-related behaviors over the summer. Given that over 5000 community-based summer day camps are available across the US, they have significant potential in helping to reduce the risk for excess summer weight gain; however, more research is needed to understand and address barriers to summer camp attendance. Future research should include a cost-effectiveness analysis to determine how summer camp compares against other obesity prevention interventions. Moreover, given the preliminary nature of the effectiveness analysis in this study, a larger randomized controlled trial that tests a nationally available summer day camp model and examines the long-term effects of summer camp on relative weight is needed.

## Data Availability

The datasets used and/or analyzed during the current study are available from the corresponding author on reasonable request.

## Abbreviations

BGC: Boys and Girls Clubs
BMIz: BMI z-score
HEI-2015: Healthy Eating Index, 2015
MVPA: Moderate to Vigorous Physical Activity
NDSR: Nutrition Data Systems for Research
SAU: Summer as Usual
SFSP: Summer Food Service Program
